# Single flap approach with or without enamel matrix derivative in the treatment of severe supraosseous defects: a retrospective study

**DOI:** 10.1007/s00784-021-03941-5

**Published:** 2021-04-14

**Authors:** Anna Simonelli, Luigi Minenna, Leonardo Trombelli, Roberto Farina

**Affiliations:** 1 Research Centre for the Study of Periodontal and Peri-Implant Diseases, University of Ferrara, Corso Giovecca 203, 44121 Ferrara, Italy; 2 Operative Unit of Dentistry, AUSL of Ferrara, Ferrara, Italy

**Keywords:** Periodontitis, Enamel matrix proteins, Surgical flaps, Regenerative medicine

## Abstract

**Aim:**

To comparatively evaluate the clinical effectiveness of the single flap approach (SFA) with and without enamel matrix derivative (EMD) in the treatment of supraosseous defects (SDs) associated with deep pockets.

**Materials and methods:**

Twenty patients, each contributing one SD associated with a deep (≥ 6 mm) pocket and treated with buccal SFA either alone (SFA group; *n* = 10) or in combination with EMD (SFA+EMD group; *n* =10), were retrospectively selected. Clinical parameters (probing depth, PD; clinical attachment level, CAL; gingival recession, REC) had been assessed at pre-surgery and 12 months post-surgery.

**Results:**

Complete wound closure was observed in 70% and 80% of defects treated with SFA and SFA+EMD, respectively. Treatments resulted in a significant PD reduction of 3.1±1.0 mm (*p*=0.005). In SFA+EMD group, 100% of closed pockets was obtained, while 90% of closed pockets was observed in SFA group. Both treatments resulted in a significant CAL gain of 2.1±0.9 mm and 1.9±1.7 mm in SFA and SFA+EMD group, respectively (*p*= 0.465). In both groups, REC significantly increased 1.0±1.1 mm in SFA group and 1.1±1.1 mm in SFA+EMD group (*p*= 0.722).

**Conclusions:**

Within their limits, the findings of present study suggest that SFA may represent a valuable option for the surgical treatment of SDs associated with deep pockets. EMD did not result in a significant clinical benefit to the procedure.

**Clinical relevance:**

SFA may represent a valuable option in obtaining pocket closure when treating SDs associated with deep residual pockets.

## Introduction

Among periodontitis-related lesions, supraosseous defects (SDs) are characterized by the coronal location of the gingival sulcus/periodontal pocket with respect to the bone crest [1]. SDs are one of the most prevalent defect types in periodontitis patients, being 3- to 9-fold more prevalent than intraosseous defects [2–4]. A radiographic study showed that untreated SDs are associated with bone loss and tooth loss within a 10-year follow-up period, although to a lesser extent compared to other defect configurations [3]. This negative prognostic impact on tooth survival may be even more relevant when the defect is combined with a bleeding pocket [5]. As a consequence, when a SD is associated with a persisting deep pocket after phase I–II of periodontal treatment, additional corrective surgical treatment is recommended [6].

SDs have been indicated as the least predictable periodontal lesion when a regenerative procedure is performed [7] and still represent a challenge for modern regenerative periodontal medicine. Their non-containing morphology does not contribute wound stability, and the wound maturation phase does not benefit from cellular support from residual lateral bony walls as in intraosseous defects [8]. Enamel matrix derivative (EMD) is a biologically active agent capable of promoting periodontal regeneration when applied on a periodontally compromised root surface after surgical debridement. In the last decade, several authors have proposed the use of EMD to promote periodontal regeneration in SDs [9–13]. A systematic review reported a weighted mean adjunctive effect of 1.2 mm on both probing depth (PD) reduction and clinical attachment level (CAL) gain for EMD over open flap debridement (OFD) at least 8 months post-surgery [14].

In 2007, the single flap approach (SFA) was proposed as a simplified surgical procedure for the regenerative treatment of intraosseous defects [15, 16]. The basic principle underlying the SFA is the elevation of a single flap (i.e., on the buccal or oral aspect only, depending on the main extension of the defect) to access the defect, leaving the interproximal supracrestal soft tissue intact [15–26]. In the regenerative treatment of intraosseous defects, SFA was associated to greater clinical results (in terms of CAL gain and intrabony component reduction) when compared to double flap approaches (DFA) based on papilla preservation techniques [27].

Based on the hypothesis that EMD may enhance the clinical outcomes of SFA surgery at SDs, the present study was performed to comparatively evaluate the clinical effectiveness of SFA with and without EMD in the treatment of SDs associated with a deep periodontal pocket.

## Materials and methods

### Experimental design and ethical aspects

The present study is a retrospective analysis of consecutively treated cases. Patients were selected among those seeking care at the Research Centre for the Study of Periodontal and Peri-implant Diseases, University of Ferrara, Italy, and one private dental office in Ferrara, Italy. All the clinical procedures were performed in accordance with the Declaration of Helsinki and the Good Clinical Practice Guidelines (GCPs). Each patient signed an informed consent to surgical treatment. The study protocol (reference code: 1096-2020-Oss-UniFe) was approved by the local Ethical Committee (Comitato Etico di Area Vasta Emilia Centro, CE-AVEC).

### Study population

Data were retrieved from the record charts of periodontal patients treated at the Research Center for the Study of Periodontal and Peri-implant Diseases (University of Ferrara, Ferrara, Italy) and a private dental office in Ferrara. For each patient contributing a SD treated with SFA+EMD (see *Clinical Procedures* for details) between January 2013 and February 2018, a patient contributing a SD treated with SFA alone was selected by matching either the highest value of pre-surgical PD (among those recorded for the mesio-buccal and mesio-palatal/lingual sites of the tooth distal to the SD, and the disto-buccal and disto-palatal/lingual sites of the tooth mesial to the SD) or the distance (evaluated during surgery) between the cementum-enamel junction and the interproximal bone crest (CEJ-BC).

Patients were included in the analysis if positive for each of the following inclusion criteria: (i) periodontal conditions compatible with a diagnosis of stage III–IV periodontitis [28, 29], as retrospectively determined on patient record charts and radiographs; (ii) undergone steps I and II of periodontal therapy [6]; (iii) undergoing SFA with or without EMD according to the description given in the paragraph “Surgical procedures”; (iv) availability of intra-operative measurements as well as clinical parameters related to pre-surgery and 2-week and 12-month follow-up visits (see “Study parameters” for details); (v) availability of a pre-surgical periapical radiograph; (vi) never smoked, former smoker, or smoker with a daily cigarette consumption ≤10 cigarettes/day; and (vii) no regular use of anticoagulants, non-steroidal anti-inflammatory drugs, corticosteroids, bisphosphonates, or biologic agents for the treatment of rheumatoid arthritis (e.g., tumor necrosis factor α, interleukin [IL]-1, or IL-6 blockers).

SDs were considered eligible for analysis when presenting the following characteristics: (i) CEJ-BC≥ 4 mm at the tooth aspects facing the defect; (ii) depth of the intrabony component ≤2 mm (as measured with a UN15 periodontal probe immediately after the completion of defect debridement); (iii) residual PD≥ 6 mm and persisting bleeding on probing (BoP) following treatment phase I–II [6]; and (iv) located to either mandibular or maxillary incisors, canines, or premolars without furcation involvement and inadequate restorations.

### Clinical procedures

#### Surgical procedures

**Fig. 1:**
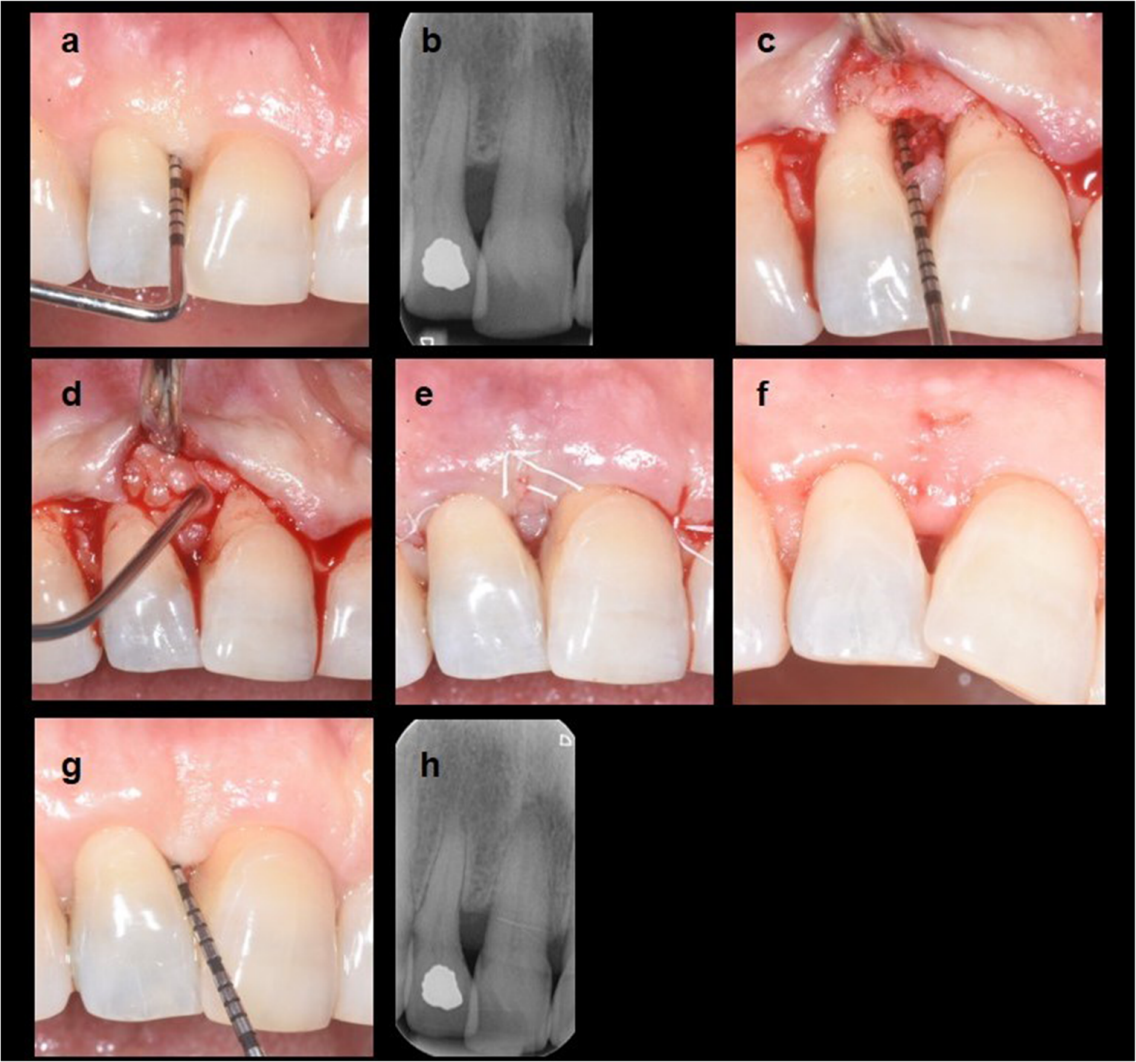
Treatment of a supraosseous defect with SFA+EMD. (**a**) supraosseous pocket (PD=8mm) at the distal aspect of the upper right second incisor, (**b**) radiographic aspect of the supraosseous lesion at pre-surgery, (**c**) clinical aspect immediately after access with a buccal SFA and surgical debridement, (**d**) EMD application after root conditioning with EDTA, (**e**) clinical aspect immediately after suturing, (**f**) complete wound closure with absence of fibrin line at the incision margins as observed at 2 weeks after surgery, (**g**) 12-month pocket closure, and (**h**) radiographical aspect at 12 months

All surgical procedures were performed by two experienced periodontal surgeons (LT, LM) by using ×2.5–3.5 magnifying loupes. Minimal modifications to the incision design of the SFA were adopted [15, 16]. Briefly, a sulcular incision was performed following the gingival margin of the teeth adjacent to the SD. The mesio-distal extension of the flap was kept as limited as possible while ensuring proper access for root and defect debridement. A horizontal, butt-joint incision was performed at the interdental papilla 1–2 mm coronal to the bone crest (as detected through pre-operative bone sounding). A buccal mucoperiosteal envelope flap was elevated by using a microsurgical periosteal elevator, leaving the residual portion of the interdental supracrestal soft tissues undetached. The root and the defect were debrided using both ultrasonic instruments and area-specific curettes. No osteoplasty was performed. EMD was applied at the operator discretion. When EMD was used (Fig. 1), the exposed root surfaces were conditioned for 2 min with 24% ethylenediaminetetraacetic acid (EDTA) and then thoroughly rinsed with saline solution. EMD was applied to the exposed root surface and alveolar crest according to the manufacturer’s instructions. Using a resorbable suture (Vicryl® 6.0, Ethicon, Sommerville, NY), a horizontal internal mattress suture was performed at the base of the papilla, and a second internal mattress suture (vertical or horizontal) was performed between the most coronal portion of the flap and the most coronal portion of the palatal/lingual papilla. Primary flap closure was always obtained at suturing.

#### Post-surgical procedures

Sutures were removed 2 weeks after surgery. The patients were asked to abstain from mechanical oral hygiene procedures in the surgical area for 4 weeks. A 0.12% chlorhexidine mouthrinse (10 mL twice daily for 6 weeks) was used to support local plaque control. Each patient was enrolled in a monthly recall program for the first 3 months and was reviewed according to personal needs thereafter. Each session included reinforcement of oral hygiene procedures and supragingival plaque removal.

### Study parameters

#### Patient-related parameters

The following patient-related parameters related to the time of the surgical procedure were extracted from the record charts: age (in years), gender, and smoking status (non-smoker/current smoker).

#### Clinical parameters

Immediately before surgery and 12 months after surgery, the following measurements were performed by using a periodontal probe with 1-mm increments (UNC15; Hu-Friedy, Chicago, IL, USA): PD, measured from the gingival margin to the bottom of the pocket; BoP, recorded as positive or negative if bleeding was detected during or after PD assessment; CAL, measured from the CEJ (or the apical margin of a restoration) to the bottom of the sulcus/pocket; and interdental gingival recession (REC), measured from the CEJ (or the apical margin of a restoration) to the gingival margin at the interproximal aspect (REC was recorded as 0 when the gingival margin was located coronal to the CEJ). PD, BoP, CAL, and REC had been evaluated at the mesio-buccal and mesio-palatal/lingual sites of the tooth distal to the SD and at the disto-buccal and disto-palatal/lingual sites of the tooth mesial to the SD. For each defect, only the site with highest pre-surgery PD among the two mesial sites and two distal sites was considered for the present analysis.

The distance between the CEJ and the interproximal bone crest (CEJ-BC), as assessed immediately after the completion of the surgical debridement in mm at the site with highest PD, was also extracted from the patient record charts.

On digital photographs taken at 2 weeks post-surgery, wound healing at the incision level was evaluated according the Early Healing Index (EHI) [30]. EHI is based on the following scale: (1) complete flap closure, no fibrin line in the interproximal area; (2) complete flap closure, fine fibrin line in the interproximal area; (3) complete flap closure, fibrin clot in the interproximal area; (4) incomplete flap closure, partial necrosis of the interproximal tissue; and (5) incomplete flap closure, complete necrosis of the interproximal tissue. EHI assessment was performed by a trained, calibrated examiner (AS).

### Statistical analysis

Data were entered into a statistical software (STATISTICA, StatSoft, Vigonza, Italy). Each patient contributed the study with one SD. If a patient presented two or more eligible SDs, one SD was randomly selected for the present study. The proportion of SDs with a 12-month PD≤4mm (i.e., *closed pockets*) was the primary outcome.

For each patient, the 12-month changes in PD, CAL, and REC were calculated by subtracting the 12-month value from the pre-surgery value. Therefore, a positive 12-month change indicates a reduction in PD and a gain in CAL or a decrease in REC. Relative CAL change (rCAL) was calculated as the percentage ratio between CAL gain and pre-surgery CAL. Data were expressed as mean ± standard deviation.

Within-group comparisons were performed with Wilcoxon test for paired observations and *χ*^2^ test or Fisher’s exact test. Inter-group comparisons were performed with Mann-Whitney *U* test and *χ*^2^ test or Fisher’s exact test. The level of significance was set at 5% for all statistical tests.

Among previous studies investigating the adjunctive effect of EMD in the treatment of SDs, only the study by Iorio-Siciliano et al. [13] reported data on the frequency distribution according to residual PD at 12 months following surgery. However, no data on the proportion of sites with 12-month PD≤ 4 mm could be retrieved from the study, thus making impossible to perform a power analysis based on the primary outcome. In order to calculate the post hoc power of the study, we performed a post hoc power calculation based on results on our primary outcome (i.e., the proportion of sites with residual PD≤ 4mm) and the size of the two treatment groups (*n*= 10). Due to the presence of a 100% value in one of the two groups, however, the calculation failed, thus making impossible to generate the post hoc statistical power of the study.

## Results

### Study population and defect characteristics

Twenty patients (10 in SFA group, 10 in SFA+EMD group), each contributing with one SD, were included. Thirteen defects had been treated (6 with SFA, 7 with SFA+EMD) at the Research Center for the Study of Periodontal and Peri-implant Diseases, University of Ferrara, Ferrara, and 7 had been treated (4 with SFA, 3 with SFA+EMD) in a private dental office in Ferrara. Patient and defect characteristics are reported in Table 1. No significant differences were found between groups for age, gender, and smoking status. Also, no inter-group difference in CEJ-BC was found.

**Table 1:** SFA and SFA+EMD groups: patient and defect characteristics

	SFA (*n* = 10)	SFA+EMD (*n* = 10)	*p*^*§*^
Patient characteristics			
Gender (males/females)	5/5	5/5	1
Age (years) (mean ± SD)	49.5 ± 8.8	52.2 ± 3.9	0.481
Smokers (yes/no)	3/7	4/6	1
Defect characteristics			
Dental arch (maxillary/mandibular)	7/3	9/1	0.582
Tooth type (referred to the tooth presenting to the site with the highest PD among the teeth adjacent to the defect) (incisor/canine/premolar)	6/2/2	7/2/1	0.833
CEJ-BC^#^ (mm) (mean ± SD) (min-max)	7.7± 1.9 (3.0–9.0)	7.9 ± 2.1 (4.5–11.0)	0.908
Infrabony component			
Present, up to 2 mm/absent	7/3	8/2	1
Mean depth (mm)	0.9 ±0.7	1.0±0.7	0.769

^#^CEJ-BC: distance between the cemento-enamel junction (CEJ) and the bone crest (BC)

§Mann-Whitney *U* test, *χ*^2^ test, or Fisher’s exact test

### Early Healing Index

Patient distribution according to EHI within each treatment group is reported in Table 2. A complete wound closure (i.e., EHI= 1–3) was observed in 7 (70%) and 8 (80%) defects treated with SFA and SFA+EMD, respectively. No significant differences in patient distribution according to EHI were found between groups (*p*= 0.959).

**Table 2:** Distribution of patients in SFA and SFA+EMD groups according to the Early Healing Index (as assessed at defect sites 2 weeks following surgery)

	SFA (*n* = 10)	SFA+EMD (*n* = 10)	*p*
Early Healing Index			
Score 1 (complete flap closure—no fibrin line in the inter-proximal area)	2	2	0.959^§^
Score 2 (complete flap closure—fine fibrin line in the inter-proximal area)	3	4	
Score 3 (complete flap closure—fibrin clot in the inter-proximal area)	2	2	
Score 4 (incomplete flap closure—partial necrosis of the inter-proximal tissue)	2	1	
Score 5 (incomplete flap closure—complete necrosis of the interproximal tissue)	1	1	

§Fisher’s exact test

### Clinical parameters

Clinical measurements as well as their 12-month changes are reported in Table 3. At pre-surgery, no significant inter-group differences in PD, CAL, and REC were observed.

**Table 3:** SFA and SFA+EMD groups: pre-surgery and 12-month probing parameters as well as 12-month changes

	Pre-surgery	12 months	*p*^*¤*^	12-month change*
PD (mm)				
SFA	6.6± 0.5	3.5± 0.7	*0.005*	3.1± 1.0
SFA+EMD	6.6± 0.7	3.5 ±0.7	*0.005*	3.1± 1.0
*p*^*¥*^	*0.898*	*0.770*		*0.968*
BoP (±)				
SFA	10/0	2/8	*< 0.001*	–
SFA+EMD	10/0	1/9	*< 0.001*	–
*p*^*¤*^	1	1		
CAL (mm)				
SFA	7.5 ± 0.7	5.4 ± 1.3	*0.005*	2.1 ± 0.9
SFA+EMD	8.3 ± 1.3	6.4 ± 2.0	*0.013*	1.9 ± 1.7
*p*^*¥*^	*0.133*	*0.282*		*0.465*
REC (mm)				
SFA	0.9 ± 1.0	1.9 ± 1.4	*0.034*	−1.0 ± 1.1
SFA+EMD	1.8 ± 1.3	2.9 ± 2.0	*0.024*	−1.1 ± 1.1
*p*^*¥*^	*0.118*	*0.298*		*0.722*

*A negative 12-month change indicates an increase in REC

¤Wilcoxon test or Fisher’s exact test

^¥^Mann-Whitney *U* test

A significant PD reduction of 3.1±1.0 mm was observed in both groups, resulting in a mean 12-month PD of 3.5 mm in either SFA + EMD or SFA group (*p*= 0.770). In SFA+EMD group, 100% of closed pockets was obtained, while 90% of closed pockets was observed in SFA group. These results were parallel by a significant reduction in the number of BoP positive sites in both groups (*p*< 0.001). Treatments resulted in a significant CAL gain of 2.1±0.9 mm (SFA group) and 1.9±1.7 mm (SFA+EMD group) (*p*= 0.465). rCAL gain amounted to 28.5 ±13.1% and 22.9 ±18.4% for SFA and SFA+EMD, respectively (*p*= 0.304).

Both treatments resulted in a similar, significant increase in REC, which amounted to 1.1±1.1mm for SFA+EMD and 1.0±1.1 mm for SFA (*p*= 0.722).

## Discussion

The presence of deep (≥6 mm), bleeding pockets after phase I and II of periodontal therapy represent a strong indication to implement periodontal therapy with additional treatments, including surgical options [6]. The present retrospective study was performed to evaluate the clinical effectiveness of a simplified surgical procedure (i.e., the SFA) with and without EMD in the treatment of SDs associated with a deep periodontal pocket persisting following phase I and II of periodontal therapy.

In the present investigation, the SFA, which was proposed for the treatment of intraosseous defects [15], has been used to access SDs. The basic principle behind the SFA is the elevation of a single mucoperiosteal flap (i.e., on the buccal or lingual/palatal side, depending on defect extension) to access the defect, and its repositioning on an undetached interproximal papilla. Substantial CAL gain and PD reduction have been reported in several prospective and observational studies on intraosseous defects treated with either SFA alone or in association with different regenerative devices [15–26]. Due to the lack of studies evaluating the effect of different flap design in the regenerative treatment of SDs [14], no clear recommendations are currently available for flap selection when surgically approaching this type of defect. Previous reports showed that at 12 months following surgery, 79.3% of SDs accessed with DFA (according to papilla preservation techniques) alone presented a residual PD of 1–3 mm [13]. In the present study, 90% of sites accessed with SFA alone showed a residual PD ≤ 4 mm. Moreover, treatment with SFA alone resulted in a CAL gain of 2.1±0.9 mm. Similar CAL gains were reported when DFA with papilla preservation alone was used to access SDs [11, 13]. Differently, markedly inferior outcomes were reported when DFA without papilla preservation (e.g., modified Widman flap) [9, 10]. Overall, these findings suggest that (1) papilla preservation techniques either with single or double flaps showed a beneficial effect in the surgical correction of SDs and (2) the SFA appears at least as effective as papilla preservation procedures to achieve pocket closure.

In our material, SFA plus EMD resulted in a CAL gain of 1.9±1.7 mm, with no significant difference with SFA group. Differently, previous studies showed that the adjunctive use of EMD significantly enhanced the outcomes of access flap when the latter was performed according to papilla preservation techniques, with a CAL gain ranging from 2.8±0.8 [11] to 3.4±0.6 mm [13]. Also, post-surgery gingival recession was less pronounced when EMD was combined with DFA [11, 13] compared to SFA+EMD group. These findings seem to indicate that DFA may represent the most appropriate surgical access to maximize the adjunctive clinical effect of EMD in SDs. However, further studies are needed to comparatively evaluate the clinical effectiveness of different flap designs when treating SDs with and without EMD. In this respect, a recent systematic review on intraosseous defects showed that the use of EMD is optimized when the lesion is surgically accessed by SFA [27].

Several studies have reported beneficial clinical effects on early soft tissue healing following the use of EMD [31–33]. In the present study, no significant differences were shown between SFA and SFA+EMD groups in terms of quality of wound healing at 2 weeks post-surgery. This finding may be explained by the fact that a complete flap closure (i.e., EHI=1, 2 or 3) was recorded in the vast majority of defects (70%) even in the SFA group (Table 2). Consistently, a large proportion of defects healed by complete closure at 2 weeks were also observed in a cohort of SFA-treated intraosseous defects [23]. These clinical observations are paralleled by experimental data showing an accelerated early wound healing phase following SFA compared to DFA [34]. Collectively, these findings suggest that the elevation of a single flap may enhance conditions for wound stability at least during the early healing phase.

The results of the present study must be considered with respect to some limitations. The latter consist mainly of the retrospective nature of the study, the lack of preliminary data to calculate the sample size a priori and the impossibility to generate the post-hoc statistical power of the study. Moreover, the lack of complementary information from radiographic exams has precluded a further analysis on radiographic bone level changes after treatment.

In conclusion, the results of the present study indicate that (i) SFA represents a valuable option in the treatment of SDs associated with presenting deep pockets and (ii) SFA+EMD do not show in a significant added benefit to the procedure in terms of CAL gain.
